# Podocyte Dedifferentiation: A Specialized Process for a Specialized Cell

**DOI:** 10.3389/fendo.2014.00148

**Published:** 2014-10-01

**Authors:** Carl James May, Moin Saleem, Gavin Iain Welsh

**Affiliations:** ^1^Academic Renal Unit, University of Bristol, Bristol, UK

**Keywords:** nephrotic syndrome, podocytes, proteinuria, epithelial–mesenchymal transition, dedifferentiation

## Abstract

The podocyte is one of the two cell types that contribute to the formation of the glomerular filtration barrier (GFB). It is a highly specialized cell with a unique structure. The key feature of the podocyte is its foot processes that regularly interdigitate. A structure known as the slit diaphragm can be found bridging the interdigitations. This molecular sieve comprises the final layer of the GFB. It is well accepted that the podocyte is the target cell in the pathogenesis of nephrotic syndrome. In nephrotic syndrome, the GFB no longer restricts the passage of macromolecules and protein is lost into the urine. A number of phenotypic and morphological changes are seen in the diseased podocyte and in the literature these have been described as an epithelial–mesenchymal transition (EMT). However, there is a growing appreciation that this term does not accurately describe the changes that are seen. Definitions of type-2 EMT are based on typical epithelial cells. While the podocyte is known as a visceral epithelial cell, it is not a typical epithelial cell. Moreover, podocytes have several features that are more consistent with mesenchymal cells. Therefore, we suggest that the term podocyte disease transformation is more appropriate.

## Introduction

The podocyte is thought to be the target cell in the pathogenesis of nephrotic syndrome. The term “podocytopathy” is being increasingly used to describe disease that has arisen due to insult or injury to the podocyte. Minimal change nephropathy (MCN), focal segmental glomerulosclerosis (FSGS), diffuse mesangial sclerosis (DMS), and collapsing glomerulopathy (CG) are all thought to be podocytopathic nephrotic diseases (1). Irrespective of the cause, podocytes demonstrate dramatic morphological differences when there is nephrotic range proteinuria (2). Actin cytoskeleton rearrangement, slit diaphragm loss, and more cuboidal morphology are all hallmark features of the diseased podocyte (2). The field has tended to refer to this loss of typical phenotype as an epithelial–mesenchymal transition (EMT) event (3–5).

However, EMT is a highly plastic and reversible process: indeed, the word “transition” was chosen deliberately to reflect this transient nature. The phenotypic and morphological changes seen in diseased podocytes are only reversible if the insult is not very severe. In minimal change disease, the disease phenotype is reversible following glucocorticoid therapy. However, in FSGS, the changes in morphology and phenotype are not only irreversible but progressive (6). Therefore, using the term EMT implies an innate reversibility, which is not strictly accurate in the case of the podocyte. Therefore, it is important to reassess the extent to which the term EMT accurately describes the transposition from the healthy to the diseased podocyte morphology. Additionally, the podocyte is not a typical epithelial cell. Despite podocytes also being known as visceral epithelial cells, they retain several mesenchymal features (spindle shaped morphology and high levels of matrix interaction) and lack archetypal epithelial markers (cell–cell contacts that are based predominantly on P rather than E-cadherin). Therefore, the podocyte fails to demonstrate the emblematic features of EMT. If the change in podocyte morphology and phenotype seen in disease does not fulfill the accepted criteria for an EMT event and is not necessarily transient in nature, then the usefulness of this term in this context must be questioned.

Immortalized podocytes *in vitro* respond to the classic inducer of EMT, TGF-B1. Following TGF-B1 treatment, human podocytes *in vitro* demonstrate increased levels of α-SMA, cadherin switch from P-cadherin to N-cadherin, and expression of the main effector transcription factors of EMT: SNAIL and SLUG (7). Similar phenotypic changes are seen in mouse podocytes *in vitro* when exposed to TGF-B1. Again suppression of P-cadherin along with suppression of ZO-1 and nephrin with concomitant upregulation of desmin, fibronectin, and collagen I is observed (8). *In vitro*, following 24 h exposure to TGF-B1, human podocytes lose their highly arborized morphology and adopt a more cobblestone-like morphology. The existence of similar phenotypic changes in both mouse and human podocytes in response to TGF-B1 is indicative of an evolutionarily conserved disease mechanism.

The pathological effects of such phenotypic changes within a cell-type, such as the podocyte, are severe. Foot process effacement is linked to a diminished ability to restrict urinary protein loss. This leads to runaway proteinuria and nephrotic syndrome. Effaced podocytes have less contact with the glomerular basement membrane, making podocyte loss much more likely. Additionally, the increased synthesis of extracellular matrix (ECM) components such as fibronectin and collagen I may contribute to GBM thickening. These are all hallmark features of diabetic nephropathy (9).

FSP1 is a fibroblast (mesenchymal) marker, which has been found in podocytes from patients with diabetic nephropathy. Healthy podocytes do not express FSP1; therefore, *de novo* expression of a mesenchymal marker is a pathological change in phenotype. Moreover, the frequency of FSP1^+^ podocytes in the urine has been linked to disease severity (8). FSP1^+^ podocytes have also been found in the glomeruli of FSGS patients (10).

The term EMT is over simplistic in this context, and does not encapsulate the process that is seen in the disease state *in vivo* and disease models *in vitro*. Broadly speaking, the morphological and phenotypic changes seen in diseased podocytes appear to be an EMT type event. However, in order for the assertion that this is an EMT event to be accurate, one must first consider to what extent the changes seen correlate with the definition of EMT.

## The Podocyte: A Specialized Cell

The podocyte is a highly specialized cell that is situated on the outer surface of the GBM. It comprises three structurally and functionally unique segments. These are known as the cell body, the major process, and the foot process (11). The cell body, the major processes, and the foot processes share a common actin cytoskeleton contractile apparatus similar to that found in smooth muscle cells or pericytes (11). The foot processes extend from the major processes and cover the GBM. Neighboring foot processes interdigitate, and where this occurs a modified cell-to-cell junction known as the slit diaphragm is formed (12). The slit diaphragm forms the final layer of the glomerular filtration barrier (GFB) and has both charge and size selective properties (13).

The foot processes are the main functional unit of the podocyte. These contain loops of filamentous actin (F-actin) that can be assembled, disassembled, and bundled together in response to the changing requirements of the foot process. The tensile strength of F-actin and its concentration in the foot processes enable the podocyte to withstand the pressure of glomerular flow (14). It is this ability of actin, to be soluble as a monomer and then rapidly polymerize to provide structure and support that allows the redistribution of the podocytes foot processes. Bivalent molecules, such as α-actinin-4 and dystrophin can link bundled actin fibers for added strength (15). There are more than 100 proteins that are involved in the regulation of actin filament formation and breakdown indicating that these are essential procedures (16). The importance of the actin cytoskeletal regulation in the proper functioning of the podocyte is indicated by the number of so-called “nephrotic” genes whose protein products act on the actin cytoskeleton (17) (Table S1 in the Supplementary Material).

Such is the importance of the podocyte and the slit diaphragm in particular that there are multiple monogenic mutations that cause nephrotic syndrome as shown in Table S1 of Supplementary Material.

Structural and functional defects in the GFB result in an inability to restrict urinary protein loss. Nephrotic syndrome is defined by the triad of proteinuria, hypoalbuminemia, and edema. Proteinuria is defined by presence of non-physiological levels of a mixture of proteins in the urine (>200 mg/l) (20). However, in clinical practice, the albumin–creatinine ratio is more likely to be employed as it accounts for differences in urine dilution, i.e., a level of >30 μg/mg demonstrates proteinuria (21). Structural and functional defects in the GFB result in an inability to restrict urinary protein loss.

Podocytes are terminally differentiated cells meaning that they are unable to proliferate. This lack of podocyte proliferation limits their capacity to recover from any damage. Therefore, podocyte injury is thought to be central to nephrotic syndrome pathogenesis. The range of podocyte injuries that can play a role in nephrotic syndrome pathogenesis is collectively referred to as podocytopathies. Many of these podocytopathies are caused by mutations in key genes. These genes are central to podocyte function; encoding either slit diaphragm proteins, transcription factors, or signaling mediators. The genetic causes of nephrotic syndrome are discussed in detail in an excellent review by Hildebrandt (17). In addition to genetic podocytopathies, there are also reactive podocytopathies. In these reactive podocytopathies, the podocyte is damaged by mediators in the microenvironment (1). These extrinsic podocyte stressors can be from several sources. They can be viral, toxic, immune-mediated, mechanical, or metabolic disorder derived.

## Epithelial–Mesenchymal Transition

It is well accepted that a loss of the highly specialized podocyte structure limits the capacity of the final layer of the GFB to restrict urinary protein loss. The changes seen in diseased podocytes could be due to an EMT event.

It has been posited in the literature that since podocytes develop from mesenchymal cells via a mesenchymal–epithelial-transition (MET) event, it is reasonable to assume that the loss of mature podocyte characteristics is the reverse of this process (22). Glomerular development consists of four stages as follows: the vesicle stage, the *S*-shaped body stage, the capillary loop stage, and the maturation stage (23). The early podocytes can first be distinguished as a layer of cells at the proximal end of the *S*-shaped body. During this stage, the podocytes develop from the columnar epithelial cells (24). In turn, the columnar epithelial cells arise from the metanephric mesenchyme via a MET event (25). At this point, the podocytes express specific podocyte markers such as Wilms’ tumor suppressor (WT1) and nephrin (26, 27). As the capillary loops form, the podocyte progenitors lose their lateral cell–cell contacts and begin to migrate. As the capillary loop stage progresses, the podocyte foot processes form. At this point, expression of the slit diaphragm proteins, nephrin, podocin, and CD2AP can be seen (28–30).

Epithelial-to-mesenchymal transition is a tightly regulated process by which epithelial cells lose their hallmark epithelial characteristics and gain the features of mesenchymal cells. EMT can be initiated in response to circulating mediators such as TGF-B1 (31, 32). During the process of EMT, the podocytes should lose their epithelial polarity, the cell-to-cell junctions (the slit diaphragm) will be altered, and the actin cytoskeleton will be rearranged (33). Following stimulation with TGF-B1, podocytes lose expression of nephrin and ZO-1 (34). As important slit diaphragm proteins, the loss of nephrin and ZO-1 expression is detrimental to the function of the podocyte. Not only does TGF-B1 lead to loss of epithelial characteristics in podocytes (as evidenced by the loss of nephrin expression) but also an increase in mesenchymal characteristics. Desmin is one such mesenchymal marker that is upregulated by TGF-B1, moreover, desmin upregulation by podocytes is seen in glomerular diseases where podocyte damage is a key feature (35).

These changes have severe consequences for the structure and function of the podocyte cell. The loss of epithelial cell–cell junctions during EMT is best represented by a loss of the slit diaphragm in the diseased podocyte. As previously described, podocytes demonstrate a clear change in their morphology and phenotype in the diseased state. Expression of the slit diaphragm is lost and the foot processes are effaced. However, does the loss of essential podocyte features seen in disease and disease models, both *in vivo* and *in vitro*, respectively, represent a typical EMT event?

Epithelial–mesenchymal transition has been categorized into three types: type 1, associated with implantation, embryo formation, and organ development, type 2, associated with wound healing tissue regeneration and organ fibrosis, and type 3, which is associated with cancer metastasis and progression (36). The distinctions between these subtypes of EMT are outside the scope of this review but are extensively covered in a review by Kalluri and Weinberg (36). For the purposes of this review, EMT will now be a reference to type-2 EMT. The criteria for type-2 EMT are listed in Table 1.

**Table 1:** **Hallmark characteristics of EMT**.

Criteria	Evidence in the literature	Criteria met?
Novel FSP1 and DDR2 expression associated with basement membrane disruption	When podocytes are exposed to high glucose concentration *in vitro*, they demonstrate a clear upregulation of FSP1 (37). Urinary podocytes from diabetic nephropathy patients are FSP1^+^ (5). Moreover, ectopic overexpression of the known EMT inducer TGF-B1 in the glomerulus stimulates FSP1 in the podocyte (38)	Partially
Increased expression of HSP47, collagen 1 (α1), collagen 2 (α2), or vimentin	HSP47 is a marker of collagen producing cells and has been found in crescentic cells but not in podocytes *in vivo* or *in vitro*. TGF-B1, a potent EMT inducer stimulates collagen 1 expression in mouse podocytes (8). Although mature podocytes express vimentin, the expression increases following TGF-B1 treatment *in vitro* (39)	Partially
Cadherin switch from E-cadherin to N-cadherin	The typical switch from E- to N-cadherin expression is not seen since mature podocytes do not express E-cadherin (40). They do, however, express P-cadherin. A switch from P-cadherin to N-cadherin is seen following TGF-B1 treatment (7)	No
Nuclear relocalization of CBF-A or B-catenin or new expression of SNAIL, SLUG, or TWIST	Nuclear translocation of beta-catenin is seen in experimental models of nephrotic syndrome both *in vitro* and *in vivo* and also in diabetic nephropathy (41). Wnt signaling is responsible for the translocation of beta-catenin and plays an important role in podocyte injury and proteinuria (41). TGF-B1 treatment stimulates SNAIL expression *in vitro*. Additionally, ectopic expression of SNAIL induces changes in podocyte phenotype consistent with EMT (8). SLUG is also expressed following TGF-B1 treatment *in vitro* (7)	Partially
Loss or reduction of epithelial cell markers	The podocyte dedifferentiation seen in response to TGF-B1 treatment is associated with a reduction in epithelial markers such as ZO-1 and P-cadherin (8, 39)	Partially
Spindle shape morphology with redistribution of stress fibers and loss of polarity	Podocytes have a spindle-like arborized morphology when fully differentiated. This morphology is lost following insult. A loss of apical–basal polarity leads to the mislocalization of nephrin and concomitant proteinuria. This loss of polarity has not been seen in models of EMT either *in vitro* or *in vivo*	No

*The dedifferentiation of podocytes seen in podocytopathic nephrotic syndromes only partially satisfies some of the criteria for a type-2 EMT transition. Clearly, the morphological and phenotypic changes seen in diseased podocytes are not obtained via a type-2 EMT event*.

The changes in morphology and phenotype between healthy and diseased podocytes only partially satisfies most of the criteria for type-2 EMT and does not fit at all with the rest. The table demonstrates quite clearly the inaccuracies of describing this process as EMT. For instance, one of the criteria for type-2 EMT is a cadherin switch from E-cadherin to N-cadherin. While the podocytes do undergo a cadherin switch in the diseased state, this is from P-cadherin to N-cadherin. No evidence could be found in the literature for podocytes expressing DDR2 or HSP47. Neither there is clear evidence for the nuclear translocation of TWIST. Otherwise, it is the lack of typical epithelial phenotype in podocytes that precludes them from matching this definition of EMT.

First of all, podocytes are atypical epithelial cells. While it is the case that podocytes demonstrate epithelial features, such as clear apical–basal cell polarity, they also exhibit mesenchymal features such as vimentin and intermediate filament expression (16). They also express features of differentiated mesenchymal cells, in particular smooth muscle actin, akin to a pericyte phenotype (42).

High migration capacity is a mesenchymal feature, while low invasive capacity is an epithelial feature, again demonstrating the dichotomous nature of the podocyte. The healthy podocyte maintains a dynamic range within which motility is regulated (43). It has been shown that plasma from patients with active FSGS significantly increases podocyte motility *in vitro* (44). The insult caused by the disease is increasing the mesenchymal characteristic of the podocyte. These hypermotile podocytes are not invasive due to the location of the podocytes on the outside of the GBM. The flux of hyperfiltrate through the GFB leads to a loss of podocytes in the urine. Indeed, as mentioned previously, viable urinary podocytes have been found with mesenchymal fibroblastic markers such as FSP1 (25).

**Figure 1:**
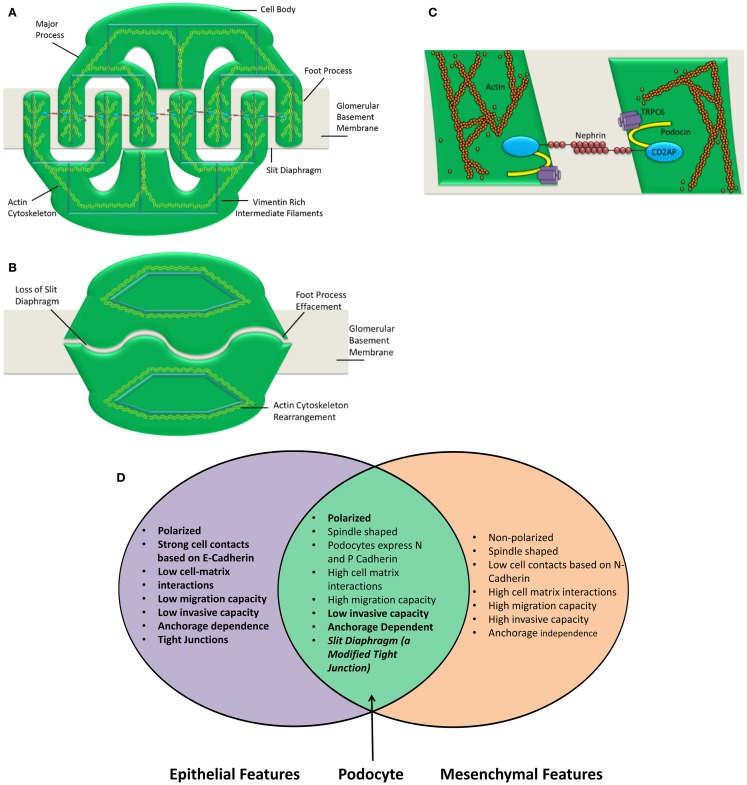
**Podocyte structure and characteristics are shown**. The podocyte comprises three main compartments, the cell body, the major processes, and the foot processes **(A)**. Each of these segments shares a common actin cytoskeleton. Neighboring foot processes regularly interdigitate. The gap between these interdigitations is bridged by a specialized cell–cell junction known as the slit diaphragm **(C)**. Following insult, the podocyte foot processes are effaced **(B)**. This concomitantly causes a loss of the slit diaphragm. The actin cytoskeleton is rearranged and the podocyte is no longer able to restrict urinary protein loss. A number of insults can cause the podocyte to lose its essential morphology rendering it unable to perform as the final layer of the glomerular filtration barrier. The number of slit diaphragms is reduced and the neat morphology of the podocyte is lost in nephrotic syndrome. This is clearly demonstrated by Patrakka et al. (68). **(D)** Epithelial and mesenchymal features of the podocyte are shown. Epithelial and mesenchymal characteristics as defined by Voulgari and Pintzas (40). The podocyte clearly possesses features that are both epithelial and mesenchymal. Based on this observation, it is not accurate to describe the podocyte as an epithelial cell. Hence, describing podocyte dedifferentiation as EMT is also an oversimplification.

Podocytes express P-cadherin instead of E-cadherin; again this is atypical for an epithelial cell (7, 45). The generation of a spindle-like morphology is indicative of a mesenchymal phenotype. However, normal podocytes both *in vitro* and *in vivo* have a highly arborized structure that is consistent with the aforementioned spindle-like morphology. Following dedifferentiation, the podocyte actin cytoskeleton rearrangement causes foot process effacement leading to the morphology seen in Figure 1. This effaced morphology could be described as analogous to the typical epithelial “cobblestone” morphology. In this way, following dedifferentiation, podocyte morphology goes from the mesenchymal-like highly arborized morphology to the epithelial-like “cobblestone” morphology. The epithelial and mesenchymal characteristics of the podocyte are summarized in Figure 1.

Therefore, when describing podocyte dedifferentiation, one must be careful to consider to what differential state the podocyte is reverting. A partial reversal of the mature, partially mesenchymal state to a more immature epithelial state seen during the *S*-shaped body, and capillary loop stages of development could be termed as MET. However, complete dedifferentiation to the “mesenchymal rest” state of development could also conceivably occur, and be more accurately described as EMT, though in neither case is the terminology adequately nuanced.

The terminology “EMT” was adapted from the original term “epithelial–mesenchymal transformation” in order to emphasize the transient nature of this mechanism and the potential for its reversal (46). Clearly, the podocyte is not the average epithelial cell and its dedifferentiation does not entirely fit with the definition of EMT outlined in this article. This is more than simply a semantic argument. Evidently, mediators such as TGF-B1 can instigate EMT and induce podocyte dedifferentiation that is at least reminiscent of EMT. However, by constraining the conceptualization of this process by only thinking in terms of EMT, novel mediators and disease processes causing podocyte dedifferentiation may be missed. In light of the criticisms of this term to describe podocyte dedifferentiation in disease as EMT, perhaps the term podocyte disease transformation (PDT) is more appropriate. With transformation reflecting that PDT is less transient than EMT.

## Genetic Forms of Nephrotic Syndrome and EMT

Single gene defects causing NS tend to cause irreversible podocyte damage. This is to be expected since many of the genes that are known to cause nephrotic syndrome express proteins that are key components of the slit diaphragm (nephrin and podocin) or are essential to the specialized architecture of the podocyte (such as alpha actinin IV). However, the incorporation of transition in this term is deliberate. The diseased podocyte phenotype can be transient. Nephrotic syndrome is often treated with glucocorticoids and/or calcineurin inhibitors both of which have been shown to have direct effects on the actin cytoskeleton of the podocyte (47–49). One key renal developmental gene, known to cause NS when mutated, has been shown to result in features of podocyte dedifferentiation. This is WT1, a transcription factor both highly expressed and necessary for renal development (50), yet only expressed in the podocyte in the mature kidney. Mouse models of the human disease resulting from WT1 mutations, Denys–Drash Syndrome (DDS), reveal loss of podocyte ZO-1 expression and upregulation of podocyte TGF-B (51). Furthermore, loss of WT1 and re-expression of PAX2 and cytokeratin in podocytes have been described in cellular lesions in FSGS biopsies, implying an epithelial switch during acquired disease (52). Our own data examining the phenotype of conditionally immortalized podocytes from children with DDS confirms re-expression of PAX2, but also clear mesenchymal features suggestive of complete dedifferentiation (unpublished results). Thus, WT1 may be a key regulator of podocyte EMT both developmentally and as a target in disease.

The extracellular domain of nephrin forms the protein scaffold of the slit diaphragm (53). There are eight IgG-like motifs in the extracellular domain each of which contains two cysteine residues that are bound to each other via disulfide bridges. In addition, there are three “free” cysteine residues per nephrin molecule that are available to form disulfide bridges with bordering nephrin molecules (54). In this way, nephrin forms the scaffold for the slit diaphragm. The extracellular domain of nephrin interacts with other proteins in order to maintain the integrity of the barrier. Two such proteins are Neph 1 and Neph 2 (55). Clearly, the loss of most of the extracellular domain, as in patients with the Fin-major mutation, has massive implications for the formation of the slit diaphragm (56). The cytoplasmic domain of nephrin, of which part is missing in patients with the Fin-minor mutation, plays a role in the maintenance of the structural and functional capabilities of nephrin (56). The cytoplasmic domain also connects nephrin, and hence the slit diaphragm to the actin cytoskeleton of the foot process (57). It has been suggested that nephrin can bind directly to the actin cytoskeleton of the foot process via its cytoplasmic domain, while Yuan and colleagues have shown that nephrin is at least capable of binding to actin (58).

Podocin comprises 383 amino acids and has a hairpin structure such that both the C and N terminus are cytoplasmic (59). It is a raft-associated constituent of the foot process membrane that is localized at the insertion of the slit diaphragm itself (29). Within the raft, podocin can form oligomers, which lead to invagination of the foot process membrane, to which CD2AP and nephrin are recruited (60). In fact, a fully functioning podocin is required for nephrin transport to the membrane (61). This is the foundation of the slit diaphragm assembly (62). Between 10 and 28% of all non-familial childhood, SRNS cases are caused by recessive podocin mutations, such is the importance of this protein (62).

CD2AP is an adaptor molecule that possesses a coiled coil domain and 3 Src homology 3 (SH3) domains (59, 63). It also has an actin binding site at its NH_2_ terminus and is believed to contribute to dynamic actin assembly (64, 65). CD2AP is capable of interacting with nephrin and in complex with nephrin and podocin is able to recruit PI3K to the plasma membrane (63).

## Conclusion

In reactive podocytopathies, the podocyte is injured by a circulating factor (66). It is clear, however, that the podocytes undergo a set of phenotypic and morphological changes during nephrotic syndrome. This process has been likened to EMT. TGF-B1 is a potent inducer of EMT. The research described in this review has relied heavily on *in vitro* work centered on the response of podocytes to TGF-B1 treatment. As demonstrated, the podocytes do not undergo a typical type-2 EMT in response to TGF-B1. The new term “podocyte disease transformation” has been coined in order to distinguish this process from EMT.

A new technique known as transcriptome *in vivo* analysis (TIVA), has been developed to analyze the transcriptome of single cell populations *in vivo* without destroying the tissue (67). This technique could be employed to further study the changes in podocytes in disease models.

The traditional definition of a podocyte as an epithelial cell is clearly simplistic, and in fact, this is a uniquely differentiated cell, fit for a specific functional purpose that fulfills features of a partial mesenchymal and partial epithelial cell. It reaches this mature phenotype via immature mesenchymal and then epithelial stages, and therefore, dedifferentiation in disease could result in regression to either of these states. It will be important in understanding podocyte disease to understand the drivers of these changes, and recognizing the developmental features that are correlated with specific clinical conditions.

## Conflict of Interest Statement

The authors declare that the research was conducted in the absence of any commercial or financial relationships that could be construed as a potential conflict of interest.

## Supplementary Material

The Supplementary Material for this article can be found online at http://www.frontiersin.org/Journal/10.3389/fendo.2014.00148/abstract
