# Supplementary material for: Podocyte Dedifferentiation: A Specialized Process for a Specialized Cell
Source: Front Endocrinol (Lausanne). 2014 Oct 1;5:148. doi: 10.3389/fendo.2014.00148 (PMC4181233; doi:10.3389/fendo.2014.00148)
Supplement: Supplementary file 1 [file Table1.PDF]

**Table S1 | Genetic causes of nephrotic syndrome**.

| Glomerular disease | Gene, protein |
|---|---|
| Congenital SRNS (Finnish type) | *NPHS1*, nephrin (SD) |
| SRNS type 2 | *NPHS2*, podocin (SD) |
| SRNS type 3 | *PLCE1* phospholipase Cε |
| SRNS type 4 | *CD2AP*, CD2-associated protein (SD) |
| Pierson syndrome | *LAMB2*, laminin β2 (BM) |
| SRNS adult onset | *NPHS2*, podocin (SD); *ACTN4*, α-ACTININ-4 (AC) |
| SRNS adult onset | *TRPC6*, transient receptor potential cation channel C6 (SD) |
| Denys–Drash syndrome, Frasier syndrome | *WT1*, WT suppressor gene (PD) |
| Glomerulopathy with fibronectin deposits | *FN1*, fibronectin (BM) |
| Alport syndrome | *COL4A5*, α5(IV)-collagen (BM) |
| Alport syndrome with leiomyomatosis | *COL4A6*, α6(IV)-collagen (BM) |
| Alport syndrome | *COL4A3*, α3(IV)-collagen (BM) |
| Alport syndrome | *COL4A4*, α4(IV)-collagen (BM) |

*SD, slit diaphragm; LS, lipid signaling; BM, basement membrane; PD, podocyte development.*

*Adapted from Hildebrandt (17). The table lists key monogenic causes of nephrotic syndrome and is not exhaustive. For a more comprehensive list, please see Ref. (18, 19). The vast majority of these genes are either integral components of the slit diaphragm or constituent parts of the glomerular basement membrane. PLCE1 is involved in lipid signaling from the membrane, ACTN4 is an actin cytoskeleton regulator, and WT1 is a key regulator of podocyte development and function.*
